# Detection of microRNA biomarkers *via* inhibition of DNA-mediated liposome fusion†

**DOI:** 10.1039/c8na00331a

**Published:** 2018-11-21

**Authors:** Coline Jumeaux, Eunjung Kim, Philip D. Howes, Hyemin Kim, Rona Chandrawati, Molly M. Stevens

**Affiliations:** Department of Materials, Department of Bioengineering, Institute of Biomedical Engineering, Imperial College London London SW7 2AZ UK m.stevens@imperial.ac.uk

## Abstract

We report the specific and sensitive detection of microRNA using an inverse DNA-mediated liposome fusion assay. This assay is homogeneous, and does not require washing, separation, or enzyme-associated amplification steps. By fine-tuning the surface functionalisation of the liposomes, liposome concentration, and assay temperature, we demonstrated a sub-nanomolar limit of detection for the target.

## Abstract 2

We report the specific and sensitive detection of microRNA using an inverse DNA-mediated liposome fusion assay.

## Graphical abstract

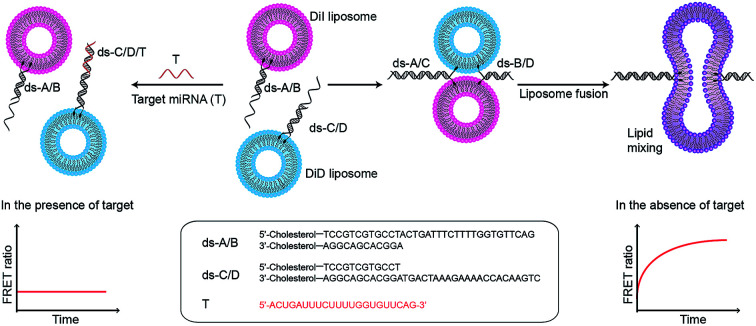

The specificity of Watson–Crick base pairing associated with sequence-directed hybridisation of nucleic acids provides a high level of control over the self-assembly of nanostructures, and this unique mechanism has inspired the use of DNA in bioengineering and biosensing applications. In particular, lipophilic nucleic acids that can insert into lipid membranes, facilitated by functional modification of the oligonucleotides with lipophilic moieties such as cholesterol, tocopherol, and porphyrin,^1,2^ have been used to mediate the assembly of lipids and liposomal constructs. DNA-controlled assembly of liposomes has notably been applied to the formation of liposome arrays on supported lipid bilayers,^3–5^ and for enabling liposome–liposome fusion (Fig. S1†).^6–9^ We recently demonstrated that DNA-mediated liposome–liposome fusion could be triggered by the specific interaction with a target microRNA (miRNA), and that this mechanism could serve as the basis of a miRNA detection assay.^10^ Liposome fusion was monitored *via* the evolution of Förster resonance energy transfer (FRET) signal (high concentrations of miRNA led to liposome fusion: high FRET, and *vice versa*). This design is successful in its own right and we hypothesise that an inverse assay, where the presence of target miRNA specifically inhibits liposome fusion, can provide a high sensitivity and a large dynamic range for a miRNA assay because the signal generated in the absence of target miRNA or at low concentrations of miRNA is high (liposome fusion: high FRET), making it easy to distinguish from the typical zero signal obtained in the absence of analytes.

Here we explore the performance of the liposome fusion inhibition mechanism at various experimental conditions, applied for the detection of miRNA-29a (miR-29a), which is involved in host response to influenza virus infection and has been recognised as a suitable biomarker for influenza.^11^ In this paper, we (i) investigate the influence of DNA functionalisation on liposomes, incubation temperature and liposome concentration to achieve optimal liposome fusion, (ii) study the sensitivity of the fusion inhibition assay at the optimised conditions for the detection of miR-29a, and (iii) evaluate the specificity of the assay against several miRNAs, belonging to the miR-29 family or specially designed to have only a few nucleobases differing from the miR-29a sequence. We further (iv) employ the developed system with separate DNA pairs to detect another target, miRNA-21 (miR-21) in complex media such as total RNA and DNA extracts. miR-21 has been associated in many types of cancers as a representative biomarker.^12^

In this assay, two populations of liposomes composed of DOPC/DOPE/cholesterol (50 : 25 : 25 mass ratio), and containing FRET donor–acceptor pairs DiI and DiD, are functionalised with complementary double stranded DNA (dsDNA) strands, dsDNA A/B (ds-A/B) and dsDNA C/D (ds-C/D) (nucleic acid sequences are detailed in ESI Table 1†), designed to hybridise in a zipper-like fashion (Fig. 1).^6,7^ The toehold region of DNA C in ds-C/D is specifically designed to be complementary to the target miRNA. In the absence of a target, ds-A/B and ds-C/D hybridise, bringing the liposomes in close contact, driving lipid mixing and allowing mixing of DiI and DiD dyes, resulting in the generation of FRET signal. When the target miRNA is present, its hybridisation to the toehold prevents ds-A/B and ds-C/D hybridising and inhibits fusion between DiI and DiD liposomes.

**Fig. 1:**
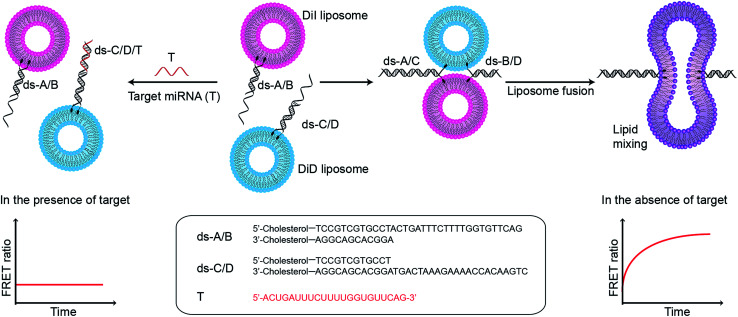
Schematic illustration of miRNA detection assay based on inhibition of DNA-mediated liposome fusion. Target miRNA (red strand, miR-29a) is complementary to the toehold on ds-C/D and hybridises to it, blocking liposome fusion. In the absence of target, liposome fusion is promoted by ds-A/B and ds-C/D hybridising in a zipper-like manner.

We assessed the extent of lipid mixing by monitoring FRET signal generation. We used the FRET pair DiI and DiD, which are hydrophobic molecules with long alkyl chains, based on Cy3 and Cy5, respectively. As lipid mixing is more efficient than full fusion,^7^ we expect enhanced signal generation when the dyes are present in the lipid bilayer rather than in the hydrophilic cavity of liposomes.

We first investigated the effect of dsDNA functionalisation on the kinetics of liposome fusion and on the efficiency of fusion inhibition after the addition of target miR-29a (Fig. 2 and S2†). Liposomes were functionalised with 100, 50, 25 or 10 dsDNA copies, then equal volumes of DiI and DiD liposomes were mixed and the FRET signal generated over time was measured. As previously reported, a higher degree of dsDNA coverage resulted in higher lipid mixing efficiency (Fig. 2).^7^ When DiD liposomes functionalised with ds-C/D were first incubated with 1 mole equivalent of miR-29a before mixing with DiI liposomes, we observed a decreased amount of lipid mixing (Fig. 2, open symbols), confirming that the presence of target was able to inhibit liposome fusion. The percentage of fusion inhibition was similar across the range of dsDNA copy numbers (Table S2†). Reducing the number of dsDNA per liposome also decreased the amount of target required to inhibit liposome fusion, which is advantageous for lowering the limit of detection of the assay.

**Fig. 2:**
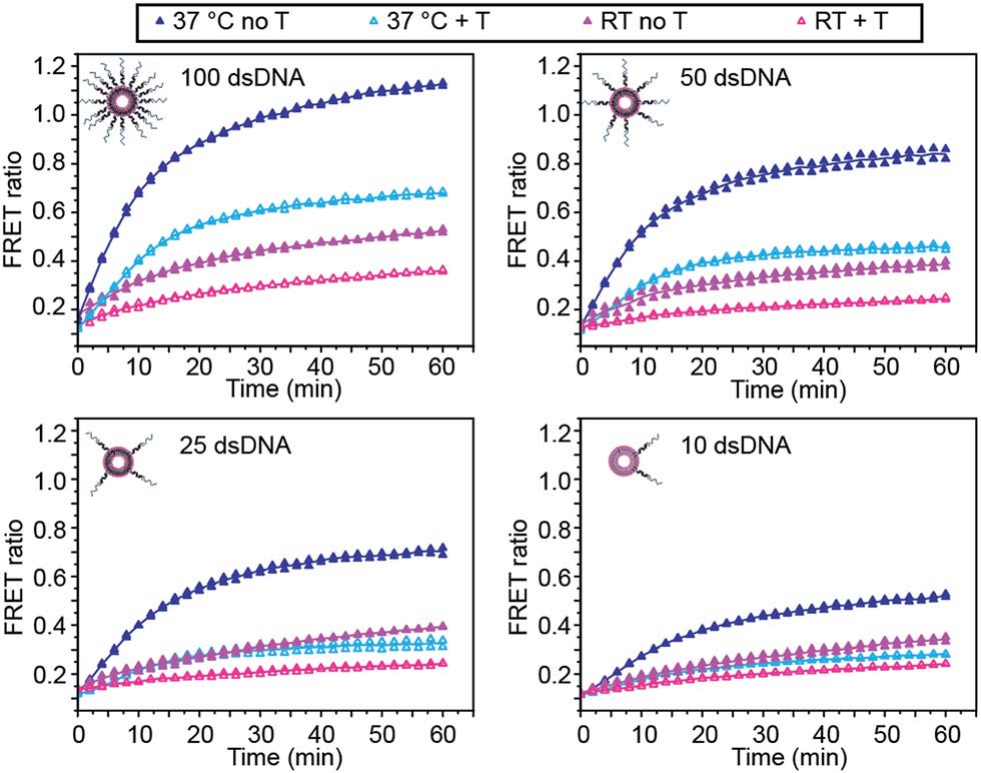
Influence of DNA functionalisation and assay temperature on the kinetics of liposome fusion. Kinetics of lipid mixing in the absence of target (closed symbols, dark) or in the presence of target miR-29a (T) (open symbols, light) for 100, 50, 25 or 10 dsDNA per liposome at room temperature (pink traces) or at 37 °C (blue traces) for two replicates. Lines represent the mean of the replicates.

We also studied the influence of assay temperature on the fusion and inhibition efficiency (Fig. 2 and S3†). Compared to room temperature, the mixture of DiI and DiD liposomes at 37 °C resulted in increased lipid mixing efficiency.^7^ When DiD liposomes were pre-incubated with miR-29a, the amount of lipid mixing was reduced, although not down to the same level as when the liposomes were mixed at room temperature.

However, the percentage of fusion inhibition notably increased when the assay was performed at 37 °C (Table S2†), which increases the dynamic margin, defined here as the difference between the FRET signals in the presence and in the absence of target. These results demonstrate the high flexibility of this assay: by varying the amount of dsDNA functionalising the liposomes and by changing the assay temperature, we have the possibility to detect a wide range of miR-29a concentrations with different dynamic margins, as well as to decrease the minimum detectable amount of the miRNA. For this study, we defined the optimal conditions for sensing of miRNA towards a low limit of detection and high dynamic margin as using liposomes functionalised with 10 dsDNA copies and incubating the liposomes at 37 °C during the assay.

We also studied the influence of liposome concentration on the rates of lipid mixing (Fig. S4†). Diluting the liposomes results in a lower abundance of dsDNA present in the solution, which in theory would require fewer target molecules to inhibit the fusion, therefore lowering the limit of detection. DiI and DiD liposomes functionalised with 100 dsDNA copies were diluted to several concentrations between 5 × 10^−4^ M and 1 × 10^−5^ M (corresponding to lipid concentration) and mixed in equal volumes. We showed that diluting the liposomes down to 1 × 10^−5^ M significantly decreased their fusion rate (Fig. S4b and c†), but the dynamic margins between the FRET ratio in the absence and in the presence of target remained unchanged upon dilution of the liposomes (Fig. S4a†). However, as the liposomes were diluted, the fluorescence signal readout decreased as well, reaching the detection limit of microplate reader. Therefore, the optimal lipid concentration used in the following assays was set at 1 × 10^−4^ M.

Having defined the optimal assay conditions, we then studied the sensitivity of this assay. DiI and DiD liposomes were functionalised with 10 dsDNA, and diluted to a lipid concentration of 1 × 10^−4^ M. DiD liposomes were incubated with various concentrations of miR-29a for 1 hour at room temperature, then equal volumes of DiI and DiD liposomes were mixed and the FRET ratio was recorded over time at 37 °C (Fig. 3a and S5†). The values of FRET ratio after 30 min of incubation at 37 °C were then plotted against miR-29a concentrations to obtain a dose–response curve (Fig. 3b). The limit of detection (LOD) was determined using the calibration curve, by first calculating the *Z* value (*Z* = blank – 3*σ*), which was converted into miR-29a concentration using the calibration curve. We obtained a LOD of 5.1 × 10^−10^ M. This result shows the strength of our assay: we were able to directly detect sub-nanomolar concentrations of miR-29a with enzyme-free signal amplification that operates at isothermal conditions. This method did not require specialised equipment and temperature cycling. Further, it reduced laborious steps and operating time without the need for specific labelling or reverse transcription of RNA into complementary DNA, which are generally required for other RNA detection methods such as polymerase chain reaction (PCR).

**Fig. 3:**
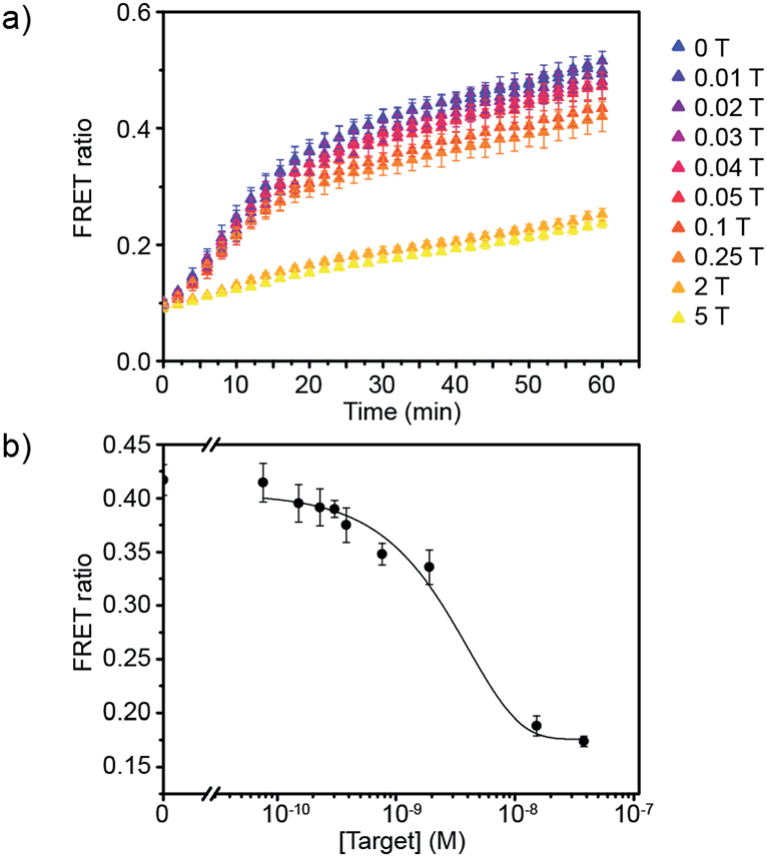
Evaluation of the sensitivity of the miRNA detection assay. (a) FRET signal traces over time in the presence of various mole equivalent of target miR-29a (1 T corresponds to 7.6 × 10^−9^ M). (b) Corresponding dose–response curve obtained after 30 min of incubating DiI and DiD liposomes together at 37 °C (liposome concentration of 1 × 10^−4^ M) (*n* = 3).

Finally, we studied the specificity of the miRNA detection assay. Members of the miR-29 family are important regulators of human diseases.^13^ miR-29a has two close relatives: miR-29b and miR-29c. Both miR-29a and miR-29b were found to be significantly differentially expressed between critically ill patients with H1N1 infection and healthy controls in a microarray experiment.^11^ In this study, we tested the ability of the assay to discriminate between the members of the miR-29 family. Additionally, as the sequences of miR-29b and miR-29c possess several nucleobase additions and mismatches compared to miR-29a, we also included in the study artificially designed miRNAs, similar in sequence to miR-29a but containing randomly mismatched 1, 2 and 3 nucleobases, respectively (ESI Table 1†). DiD liposomes were incubated with the different miRNAs at a final concentration of 7.6 × 10^−9^ M. Then, an equal volume of DiI liposomes was added and the kinetics of lipid mixing were measured at 37 °C (Fig. 4a and S6†). The values of the FRET ratio relative to the FRET ratio of the negative control (liposomes mixed in absence of target) after 30 min of incubation for each miRNA are reported in Fig. 4b. miR-29a showed the highest degree of fusion inhibition, followed by miR-29a 1MM and miR-29a 2MM. The rest of the miRNAs tested did not significantly differ from the negative control at the concentration tested. As miR-29a 1MM only differs by one nucleobase from miR-29a, we expect it to be able to hybridise to the toehold and to partially inhibit liposome fusion, as was observed in this experiment. While the inhibition of fusion by miR-29a 1MM and miR-29a 2MM is significantly lower than for miR-29a, we postulate that the incomplete specificity towards miR-29a can be problematic in a complex mixture of miRNAs, where it may not be possible to resolve if the signal corresponds to solely miR-29a or various combinations of miR-29a, miR-29a 1MM and miR-29a 2MM. In comparison, our other DNA-mediated liposome fusion assay based on hairpin displacement had higher specificity, however at the expense of design simplicity.^10^

**Fig. 4:**
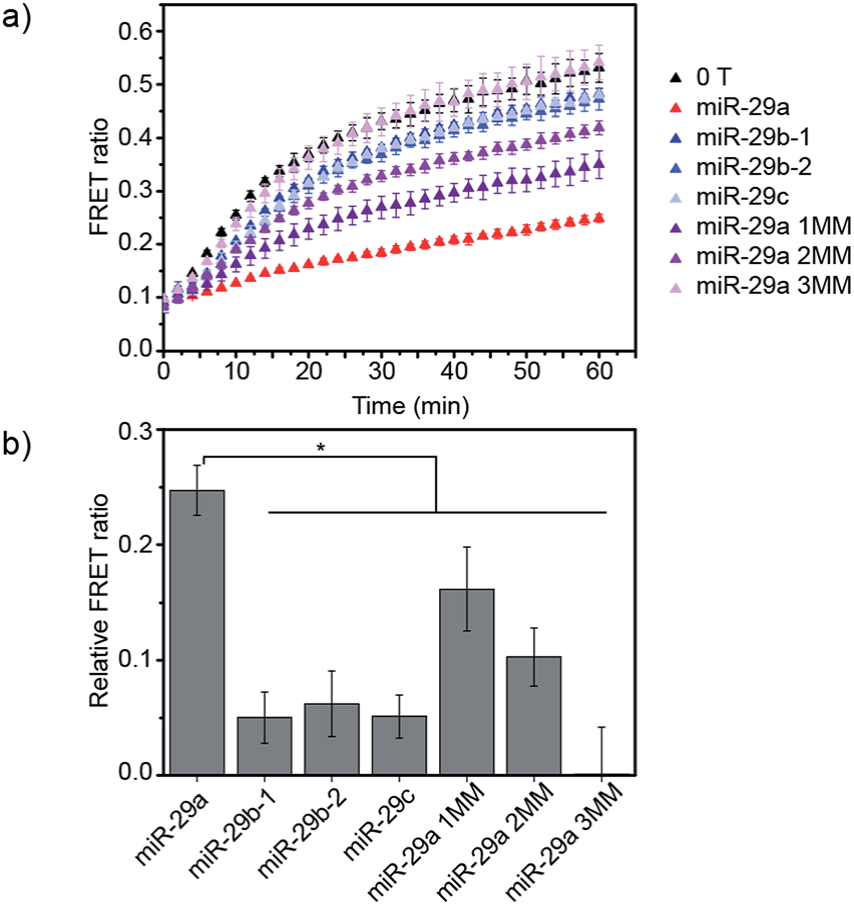
Evaluation of the specificity of the miRNA detection assay. (a) FRET signal traces over time in the presence of various miRNA sequences added to DiD liposomes at a concentration of 7.6 × 10^−9^ M. (b) Corresponding bar chart representing the FRET ratio value for each miRNA sequence after 30 min of mixing DiI and DiD liposomes at 37 °C (liposome concentration of 1 × 10^−4^ M) (*n* = 3, *p < 0.05 (ANOVA followed by Tukey *post-hoc* test)).

We further evaluated the detection capability of the assay in the presence of a pool of other RNA or DNA molecules to mimic the complexity of biological samples. We chose miR-21 as another target, which has been identified as a key biomarker associated with diverse cancers.^12^ Total RNA and DNA extracts were isolated from human kidney embryonic HEK 293T cells in which the miR-21 expression is downregulated compared to that in cancer cells.^14,15^ Different concentrations of synthetic miR-21 were spiked in the assay buffer containing 100 ng of total RNA or genomic DNA extracts at final concentration of 1.25 μg mL^−1^. We showed the FRET signal changes over time for the spiked miR-21 samples with their dose response curves (Fig. 5). The estimated LODs were 3.9 × 10^−9^ M, 3.6 × 10^−9^ M, and 2.6 × 10^−9^ M for the spiked samples in assay buffer, total RNA, and genomic DNA extracts. With even higher amounts of RNA extract (500 ng), we did not see significant variations in the detection limit of our assay (Fig. S7†). These results confirm that the assay is capable of analysing various miRNAs by employing the corresponding dsDNA pairs and selectively detecting target miRNA in complex media.

**Fig. 5:**
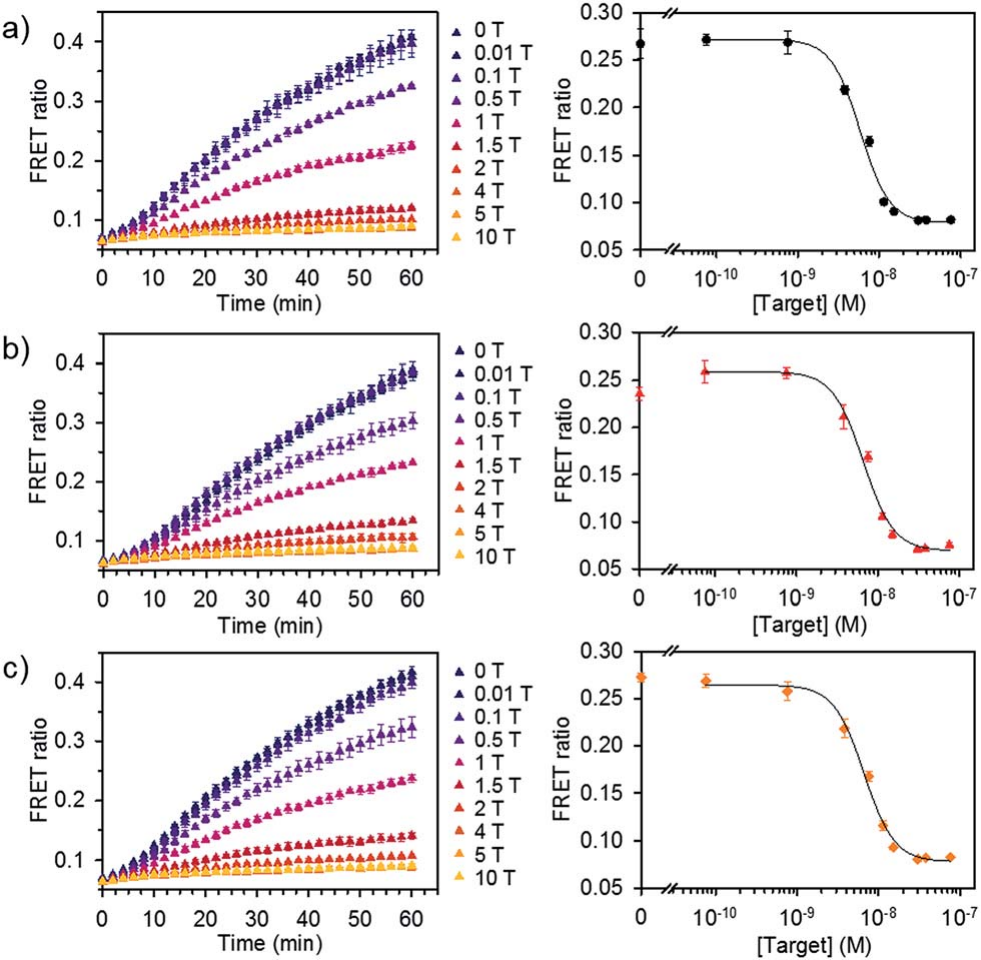
Detection of miRNA spiked in total RNA and DNA extracts. FRET signal traces over time (left) and the corresponding dose–response curve (right) obtained from (a) assay buffer, (b) diluted total RNA, and (c) genomic DNA extracts spiked with various concentrations of miR-21. Dose–response curves were obtained after 30 min of incubating DiI and DiD liposomes together at 37 °C (liposome concentration of 1 × 10^−4^ M, 1 T corresponds to 7.6 × 10^−9^ M) (*n* = 3).

In this study we have demonstrated the detection of miRNA using a simple and rapid enzyme-free diagnostic platform, based on the inhibition of DNA-mediated liposome fusion. The assay is sensitive to miR-29a at sub-nanomolar concentrations (5.1 × 10^−10^ M), and is specific towards the nucleic acid sequence of miR-29a. Furthermore, the assay is versatile for various panels of miRNAs, as shown by the assay performance to detect miR-21 in cellular RNA and DNA samples. This work demonstrates the potential of using interactions with target molecules to mediate the liposome fusion process, and of achieving a high degree of sensitivity and selectivity for biosensing applications using such an approach. We anticipate that further tuning of liposome composition, surface-bound DNA fusion mechanisms, and fluorescent reporter molecules will lead to even greater sensitivity, and that this mechanism has potential for use in disease diagnostics targeting miRNA and other nucleic acids. Furthermore, we postulate that the working principle of our assay can be extended to the detection of small molecules or proteins such as adenosine-5′-triphosphate (ATP), thrombin, or prostate-specific antigen (PSA) as the DNA aptamer sequences targeting these molecules can be also encoded in the toehold region.

## Conflicts of interest

The authors declare no conflict of interest.

## Supplementary Material

NA-001-C8NA00331A-s001
