# Supplementary material for: Detection of microRNA biomarkers via inhibition of DNA-mediated liposome fusion
Source: Nanoscale Adv. 2018 Nov 21;1(2):532–6. doi: 10.1039/c8na00331a (PMC9473185; doi:10.1039/c8na00331a)
Supplement: NA-001-C8NA00331A-s001 [file NA-001-C8NA00331A-s001.pdf]

Electronic Supplementary Material (ESI) for Nanoscale Advances.
This journal is © The Royal Society of Chemistry 2018

Electronic Supplementary Information

Detection of microRNA biomarkers via inhibition of DNA-mediated liposome fusion

Coline Jumeaux,[a,∞] Eunjung Kim,[a,∞] Philip D. Howes,[a,¶] Hyemin Kim,[a] Rona Chandrawati,[a,§] and Molly M. Stevens[a,*]

[a] Department of Materials, Department of Bioengineering, and Institute of Biomedical Engineering, Imperial College London, London, SW7 2AZ, UK.

E-mail: m.stevens@imperial.ac.uk

[∞] These authors contributed equally.

Present addresses:

[¶] Department of Chemistry and Applied Biosciences, ETH Zürich, CH-8093 Zürich, Switzerland.
[§] School of Chemical Engineering, The University of New South Wales (UNSW Sydney), Sydney, NSW 2052, Australia.

Experimental section

Materials: 1,2-Dioleoyl-sn-glycero-3-phosphocholine (DOPC) and 1,2-dioleoyl-sn-glycero-3-phosphoethanolamine (DOPE) lipids were purchased from Avanti Polar Lipids, Inc. Cholesterol, tris(hydroxymethyl)-aminomethane hydrochloride (Tris), sodium chloride (NaCl), and ethylenediaminetetraacetic acid (EDTA) were obtained from Sigma-Aldrich. 1,1′-Dioctadecyl-3,3,3′,3′-tetramethylindodicarbocyanine perchlorate, DiIC18(5) (DiD) and 1,1′-dioctadecyl-3,3,3′,3′-tetramethylindocarbocyanine perchlorate, DiIC18(3) (DiI) dyes were purchased from Invitrogen Life technologies. Oligonucleotides were purchased from IBA GmbH (Germany), Integrated DNA Technologies (Belgium), and Eurogentec (Belgium) (Table S1). Nuclease-free water (non DEPC-treated) was purchased from Thermo Fisher Scientific (Ambion™).

Liposome preparation and functionalisation: Liposomes were composed of DOPC/DOPE/Cholesterol (50:25:25 mass ratio) and contained 2 mol % of DiI or DiD. Dry lipid films were hydrated in TE buffer (10 mM Tris, 1 mM EDTA, pH 8.0) containing 150 mM NaCl at 37 °C for 30 min. Liposomes were extruded using the Avanti Lipid Mini-Extruder with 100 nm polycarbonate membranes (Whatman). Each DNA strand (A and B, C and D) was mixed at 1:1 molar ratio in TE buffer (10 mM Tris, 1 mM EDTA, pH 7.9) containing 50 mM NaCl. To form the DNA duplexes, the mixtures were annealed at 80 °C for 5 min, then allowed to hybridise by slowly cooling down the solution to 25 °C. DiI liposomes were functionalised with ds-A/B and DiD liposomes were functionalised with ds-C/D by allowing the cholesterol modified double-stranded DNA (dsDNA) to self-incorporate in the liposomes for 1 h. Unbound DNA was removed using Nanosep® Centrifugal Device with Omega Membrane (MWCO 30 kDa, 5 min × 5000 g, Pall).

Measurement of fusion kinetics: Liposomes were diluted to the required concentration in TE buffer containing 150 mM NaCl (pH 7.4). Various concentrations of target miR-29a (for evaluation of the sensitivity of the assay) or various other miRNAs (for evaluation of the specificity of the assay, see Supporting Table S1 for the detailed nucleic acids sequences) were added to solutions of DiD liposomes, and allowed to hybridise for 1 h at room temperature. For evaluation of the specificity, the concentration of miRNA was set to 7.6×10^{-9} M (1:1 molar equivalent). Then, equal volumes of DiI and DiD liposomes were mixed. Kinetics of fusion of DiI and DiD liposomes were measured in 96-well plates, and fluorescence emission of the dyes was recorded with a SpectraMax M5 microplate reader (Molecular Devices, USA) at 25 °C or 37 °C, using λ_{ex} = 530 nm, $_{\lambda em,DiI}$ = 570 nm, $\lambda_{em,DiD}$ = 670 nm.

Determination of the sensitivity and specificity of the assay: DiI and DiD liposomes were functionalised with 10 dsDNA copies per liposome, and liposomes were diluted in TE buffer containing 150 mM NaCl (pH 7.4) to 1×10^{-4} M (lipid concentration based on the total lipid and cholesterol concentration prior to extrusion). The assay temperature was 37 °C. Results were obtained in triplicate.

Cell culture and total RNA and DNA extraction: Human embryonic kidney cells HEK 293T were purchased from ATCC. Cells were maintained in Dulbecco's modified essential medium (Gibco, Thermo Fisher Scientific) supplemented with 10% (v/v) fetal bovine serum (Gibco) and 1% (v/v) penicillin/streptomycin (Gibco) for 72 h before harvest. Total RNA, including miRNA and mRNA, was isolated with the miRNeasy Mini Kit (Qiagen), and genomic DNA (gDNA) was isolated with the

AllPrep DNA/RNA Mini Kit (Qiagen) according to the manufacturer's instructions. After purification, the total RNA and gDNA were eluted with nuclease-free water. Their quantity and quality were determined by a NanoDrop 2000c spectrometer (Thermo Fisher Scientific). The RNA and DNA samples were kept at -80 °C for further use.

Detection of miRNA in a mixture of RNA or DNA: For miR-21 detection, the toehold region of DNA C in ds-C/D was designed to be complementary to the target miR-21, and DNA A was designed accordingly (see Supporting Table S1 for the detailed nucleic acids sequences). Various concentrations of miR-21 were spiked into TE buffer containing 150 mM NaCl (pH 7.4) with 100 ng or 500 ng of total RNA or 100 ng of gDNA extracts. The spiked miRNA was added to solutions of DiD liposomes, followed by incubation for 1 h at room temperature. After mixing with equal volumes of DiI liposomes, the kinetics of fusion were performed in the same procedures as described above. DiI and DiD liposomes functionalised with 10 dsDNA copies per liposome at the lipid concentration of 1 × 10^{-4} M were used. The assay temperature was 37 °C. Results were obtained in triplicate.

Supplementary tables and figures

Table S1. Nucleic acid sequences used in this study.

Name	Sequence (5’ – 3’)	Modification	Provenance
A for miR-29a	TCCGTCGTGCCTACTGATTTCTTTTGGTGTTCAG	5’-Cholesterol	IBA GmbH
A for miR-21	TCCGTCGTGCCTTAGCTTATCAGACTGATGTTGA	5’-Cholesterol	Eurogentec
B for both miRNAs	AGGCACGACGGA	3’-Cholesterol	IBA GmbH
C for miR-29a	CTGAACACCAAAAGAAATCAGTAGGCACGACGGA	3’-Cholesterol	IBA GmbH
C for miR-21	TCAACATCAGTCTGATAAGCTAAGGCACGACGGA	3’-Cholesterol	Eurogentec
D for both miRNAs	TCCGTCGTGCCT	5’-Cholesterol	IBA GmbH
miR-29a Target	ACUGAUUUCUUUUGGUGUUCAG	-	IBA GmbH
miR-29b-1	GCUGGUUUCAUAUGGUGGUUUAGA	-	IDT
miR-29b-2	CUGGUUUCACAUGGUGGCUUAG	-	IDT
miR-29c	UGACCGAUUUCUCCUGGUGUUC	-	IDT
miR-29a 1MM	ACUGAUUUCUUCUGGUGUUCAG	-	IDT
miR-29a 2MM	ACUGAGUUCUUCUGGUGUUCAG	-	IDT
miR-29a 3MM	ACUGAGUUCUUCUGGUGCUCAG	-	IDT
miR-21 Target	UAGCUUAUCAGACUGAUGUUGA	-	IDT

Nucleobases binding to miR29-a and miR-21 are underlined

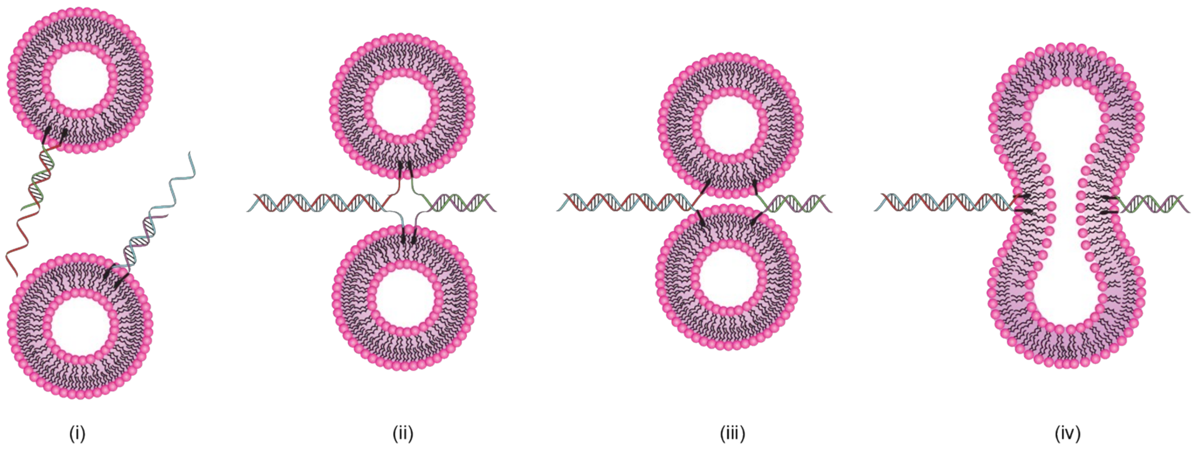

Fig. S1. Mechanism of DNA-mediated liposome fusion. (i) Two separate populations of liposomes are functionalised with complementary DNA strands; (ii) and (iii) DNA hybridisation in a zipper-like fashion brings two liposomes in close contact, eventually leading to mixing of outer leaflet lipids, (iv) and in some cases opening of the fusion pore.

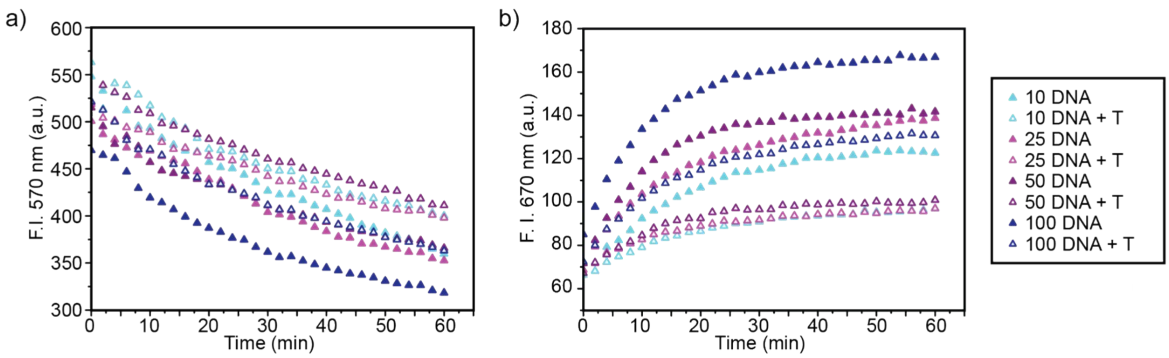

Fig. S2. Kinetics of fusion at room temperature of lipid vesicles functionalised with 10 (cyan), 25 (pink), 50 (purple) or 100 (blue) DNA per liposome on average, in the absence (closed symbols) or in the presence (open symbols) of target miR-29a ([miR-29a] = 7.6 × 10^{-9} M). a) Fluorescence emission at 570 nm, and b) Fluorescence emission at 670 nm. Data represents means of n = 2.

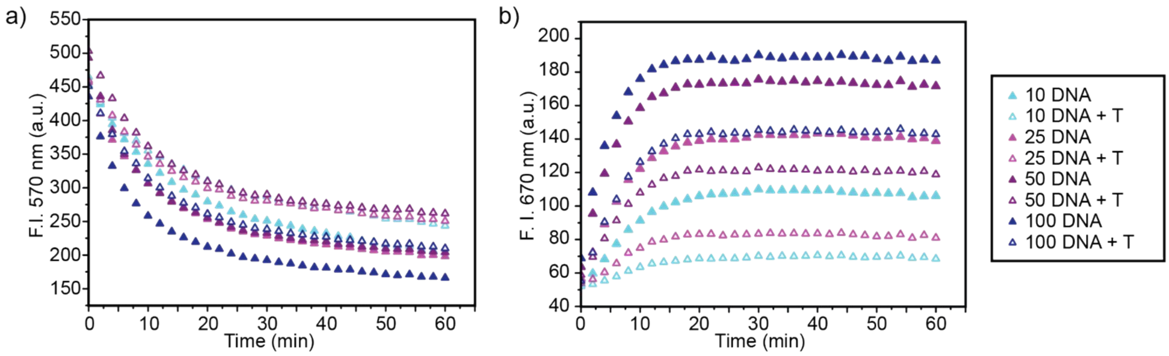

Fig. S3. Kinetics of fusion at 37 °C of lipid vesicles functionalised with 10 (cyan), 25 (pink), 50 (purple) or 100 (blue) DNA per liposome on average, in the absence (closed symbols) or in the presence (open symbols) of target miR-29a ([miR-29a] = 7.6 ×1 0^{-9} M). a) Fluorescence emission at 570 nm, and b) Fluorescence emission at 670 nm. Data represents means of n = 2.

Table S2. Percentage of fusion inhibition (F_{inib}) after 60 min of mixing DiI and DiD liposomes, calculated as $F_{inib} = 1 - (FRET_{+target} / FRET_{-target})$ where $FRET_{+target}$ is the FRET value in the presence of target and $FRET_{-target}$ is the FRET value in the absence of target.

	Number of dsDNA per liposome on average			
Assay temperature	10	25	50	100
RT	29 %	38 %	37 %	31 %
37 °C	46 %	54 %	46 %	39 %

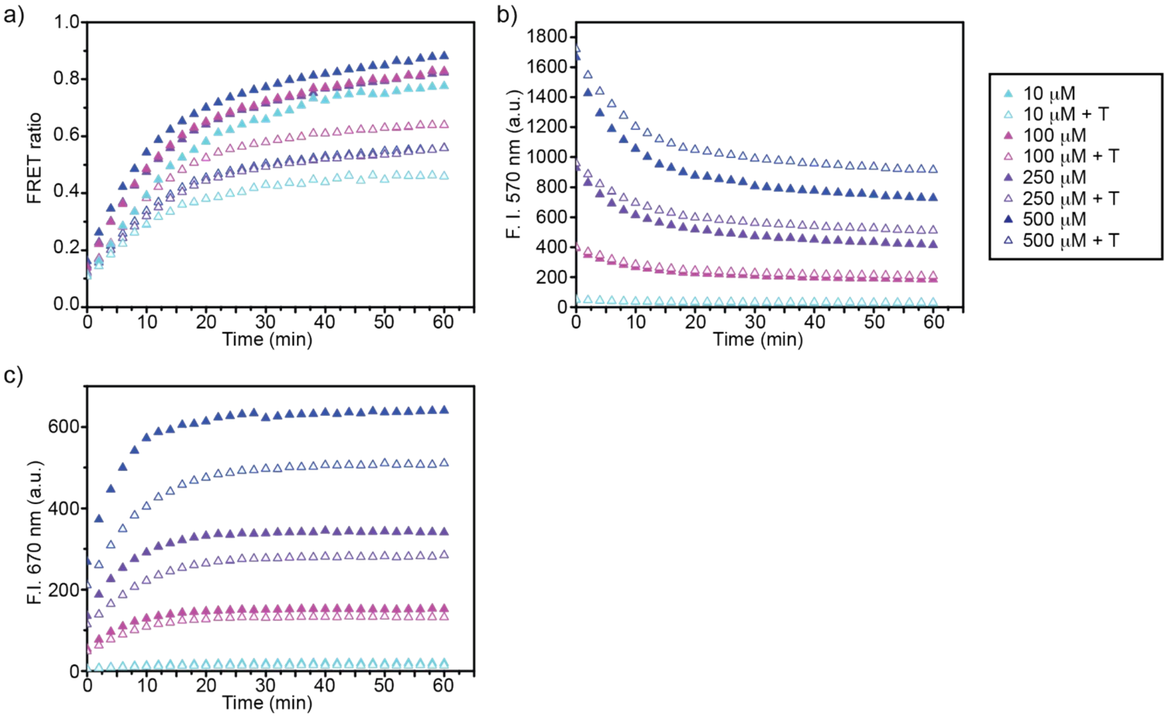

Fig. S4. Study of the influence of lipid vesicle concentration on the assay. Kinetics of fusion at 37 °C of lipid vesicles diluted to 10 µM (cyan), 100 µM (pink), 250 µM (purple) or 500 µM (blue), in the absence (closed symbols) or in the presence (open symbols) of target miR-29a, introduced at 1:1 molar equivalents. Lipid concentrations are derived the initial lipid and cholesterol concentration prior to extrusion. a) FRET ratio, b) Fluorescence emission at 570 nm (NB: the trace for 10 µM lies directly underneath the 10 µM + T), c) Fluorescence emission at 670 nm. Data represents means of n = 2.

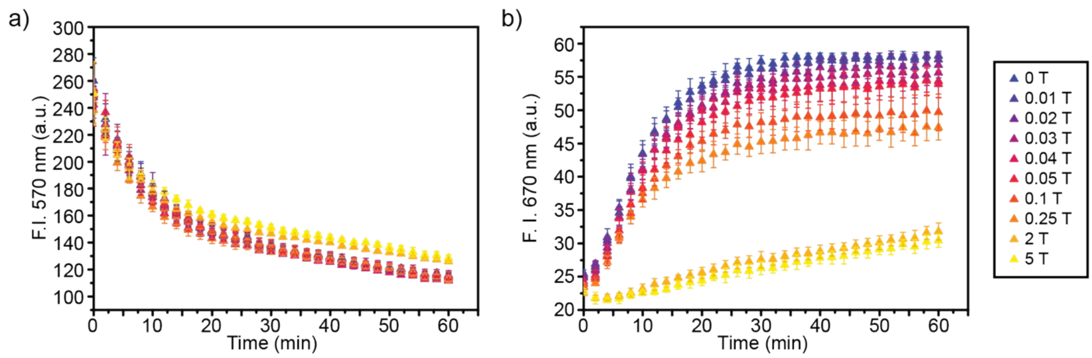

Fig. S5. Evolution of fluorescence emission at a) 570 nm, and b) 670 nm when exciting at 530 nm, showing the kinetics of fusion in the presence of different quantities of miR-29a (where 1 T corresponds to 7.6×10^{-9} M) (n = 3). Error bars represent standard deviation.

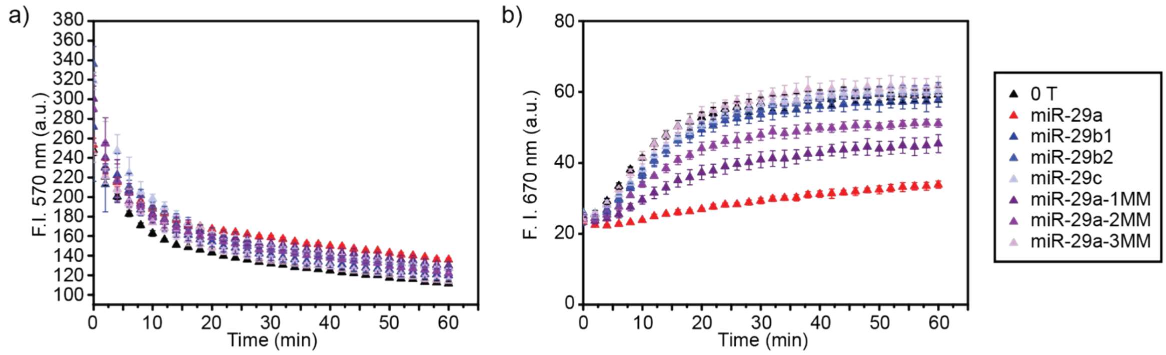

Fig. S6. Evolution of fluorescence emission at a) 570 nm, and b) 670 nm when exciting at 530 nm, showing the kinetics of fusion in presence of various miRNA sequences added to DiD liposomes at a concentration of 7.6×10^{-9} M (n = 3). See Table S1 for detail of the sequences. Error bars represent standard deviation.

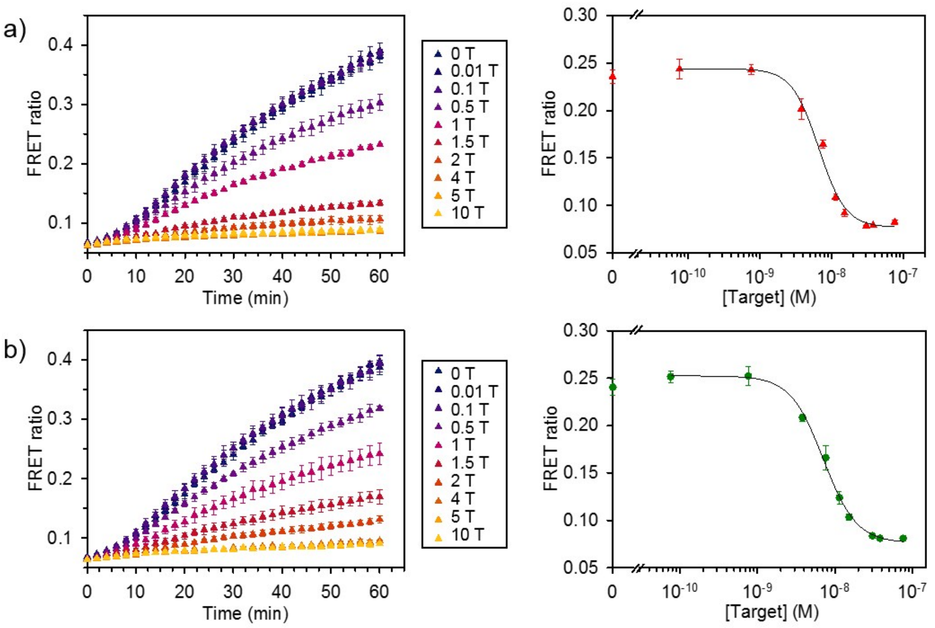

Fig. S7. FRET signal traces over time (left) and the corresponding dose-response curves (right) of various concentrations of miR-21 spiked with assay buffer containing a) 100 ng and b) 500 ng of total RNA extracts. The estimated limit of detections were 3.5×10^{-9} M and 3.7×10^{-9} M, respectively. Dose-response curves were obtained after 30 min of incubating DiI and DiD liposomes together at 37 °C (liposome concentration of 1×10^{-4} M, 1 T corresponds to 7.6×10^{-9} M) (n = 3). Error bars represent standard deviation.
